# Tubularization of Bone-Tendon-Bone Grafts: Effects on Mechanical Strength and Postoperative Knee Stability in Anterior Cruciate Ligament Reconstruction

**DOI:** 10.3390/medicina59101764

**Published:** 2023-10-03

**Authors:** Mirko Obradović, Srđan Ninković, Nemanja Gvozdenović, Milan Tošić, Milan Milinkov, Oliver Dulić

**Affiliations:** 1 Clinic for Orthopedic Surgery and Traumatology, Clinical Center of Vojvodina, 21000 Novi Sad, Serbia; srdjan.ninkovic@mf.uns.ac.rs (S.N.); nemanja.gvozdenovic@mf.uns.ac.rs (N.G.); 911025d22@mf.uns.ac.rs (M.T.); 014293@mf.uns.ac.rs (M.M.); oliver.dulic@mf.uns.ac.rs (O.D.); 2 Faculty of Medicine, University of Novi Sad, 21000 Novi Sad, Serbia

**Keywords:** anterior cruciate ligament reconstruction, bone-tendon-bone graft, animal model, biomechanical properties, clinical study

## Abstract

*Background and Objectives:* The study addresses a significant limitation in applying bone-patellar tendon-bone (BTB) grafts in anterior cruciate ligament (ACL) surgery. By exploring the tubularization of grafts, the study extends the understanding of this surgical technique. The dual approach of the study—focusing on biomechanical properties using an animal model and postoperative outcomes in humans—offers a comprehensive perspective. *Materials and Methods:* The experimental cohort encompassed ten pairs of fresh porcine bone-tendon-bone grafts. One graft in each pair underwent modification through sutures that transformed the flat graft into a cylindrical structure. Testing determined the force required for the modified graft to rupture mechanically, expressed as N/mm^2^, compared to conventionally prepared bone-tendon-bone grafts. The second phase of the research involved a prospective randomized clinical trial comprising 120 patients undergoing operative ACL reconstruction. For half the cases, grafts were tubularized using a random selection process. Clinical evaluations preoperatively and 12 months postoperatively employed the Tegner, Lysholm, and IKDC scoring scales for knee assessment. *Results:* Experiments showed that ligaments made using the tubularized surgical technique have statistically significantly higher values of measured force and higher maximum elongation values than ligaments made using the classical method. The clinical study concluded that there was no significant difference between the two groups of patients in the average score on the Tegner, Lysholm, and IKDC scales before and after surgery. *Conclusions:* The study results showed that suturing the graft does not negatively affect its biomechanical properties, and tubularization significantly increases the values of force required to cause rupture and the values of maximum elongation during rupture. Given the possibility of the one-year follow-up period being insufficient, future investigations should extend this period to acquire objective functional insights post-surgery.

## 1. Introduction

Anatomical reconstruction of the anterior cruciate ligament (ACL) is a critical goal in restoring the natural knee anatomy. Factors, including the graft position, mechanical characteristics, fixation strength, and biological response, must be considered to achieve optimal results [1,2,3]. Notably, the size of the graft, rather than the size of the native ACL attachment site, determines the size of the reconstructed ACL [4]. Understanding the geometric characteristics of the graft, such as length and cross-sectional size, is crucial for ensuring optimal mechanical properties and successful reconstruction.

In the initial stages after implantation, the strength of the graft plays a crucial role in revascularization and recollagenization. However, during this early phase, there is a significant loss of graft strength, sometimes exceeding 50% [5]. Therefore, the initial strength of the graft should surpass that of the tissue being replaced. Clinical studies have shown that the process of ligamentization is similar to that in animals, but the remodeling process is prolonged in humans [6,7,8].

Among graft options, bone-patellar tendon-bone (BTB) grafts offer numerous advantages, including strength, stiffness, high maximum load, strong fixation, consistent dimensions, and simple surgical access [9,10]. However, BTB grafts also have certain drawbacks, such as lower values of cross-sectional area relative to the bone tunnel and incomplete filling of the tunnel with the graft [11,12,13].

Various studies have investigated the cross-sectional area of autografts from the patellar ligament. Intraoperative measurements and postoperative magnetic resonance imaging (MRI) evaluations have shown that the cross-sectional area of patellar ligament autografts is around 33 mm^2^, with an average value of 36 mm^2^ calculated in a cadaveric study [14,15]. In comparison, the cross-sectional area of the native ACL ranges from 32 to 65 mm^2^, as confirmed in cadaveric studies [16].

Several attempts have been made to enhance the BTB graft technique. While some have focused on designing quadrangular tunnel and bone block grafts [17,18,19], others have tried longitudinally splitting bone blocks and folding cadaveric grafts to increase the graft’s cross-sectional area [20].

Recently, a novel graft preparation method was introduced, which employs sutures to convert a flat tendon into a tubular structure, effectively tubularizing the graft [21]. This innovative design is grounded in the belief that a tubularized structure can potentially mimic the natural rounded shape of the ACL, aiming to increase its cross-sectional area. The scientific rationale behind this design considers that a tubular structure might distribute the load more evenly across the graft, thereby potentially improving its biomechanical stability and longevity. Advantages of this approach include a closer match to the native ACL’s biomechanical properties, possibly enhanced graft integration, and reduced risk of graft impingement. However, potential drawbacks might consist of complexities in the surgical procedure and uncertainties about long-term outcomes.

The subject of this study is to modify the technique of preparing BTB grafts to increase its cross-section, enhancing its mechanical strength and providing a more suitable ligamentization process.

## 2. Materials and Methods

### 2.1. Objectives of This Research Were

1. To determine, using an animal model, whether tubularization of bone-tendon-bone autografts affects the force required for mechanical rupture of the graft;
2. To determine whether tubularization of bone-tendon-bone autografts affects postoperative knee stability;
3. To compare the obtained results with clinical results of arthroscopic reconstruction of the anterior cruciate ligament using an identical surgical procedure performed without tubularization of bone-tendon-bone autografts.

### 2.2. The Experimental Part of the Research

**Figure 1:**
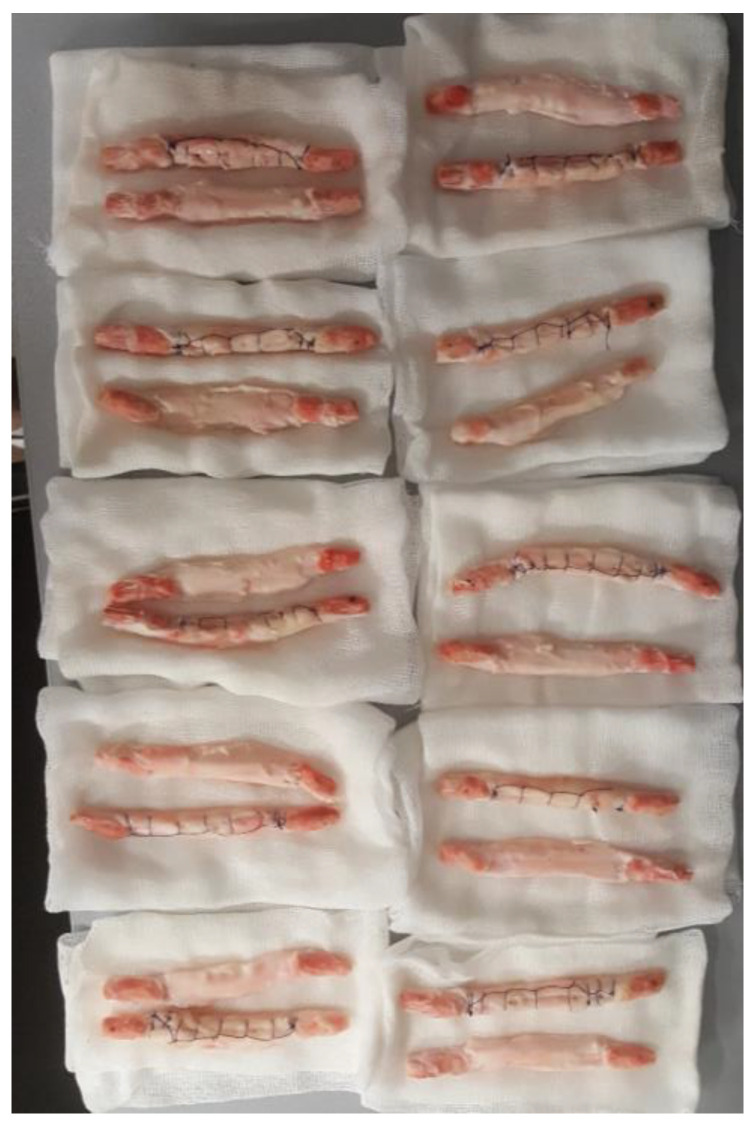
10 pairs of fresh porcine bone-tendon-bone grafts.

The experimental part of the research was conducted at the Faculty of Technical Sciences (FTN) in Novi Sad, at the Department of Production Engineering. The testing was performed on a mechanical testing machine (WPM-Rauenstein, Universal Testing Machine, Leipzig, Germany). The experimental material consisted of 10 pairs of fresh porcine bone-tendon-bone grafts (Figure 1). Each pair consisted of BTB grafts taken from the hind legs of the same pig of the Yorkshire breed, aged 6–8 months. One of the grafts in each pair was modified using sutures that converted the tendon into a cylindrical structure.

To ensure uniformity and consistent results across all specimens, the following precautions were taken:

Selection Criteria: Only grafts that met a predetermined size and weight range were selected for the study. This ensured that the anatomical variances were minimal.

Graft Preparation: A standardized protocol was employed for graft extraction, storage, and preparation, ensuring consistent anatomical and physiological conditions across the grafts.

Tubularization Process: The technique for tubularizing the tendon with sutures was standardized. A specific type, size, and pattern of sutures were used, and the same number of turns and knots was applied to each graft. This protocol ensured that each tendon achieved a consistent cylindrical shape and size.

**Figure 2:**
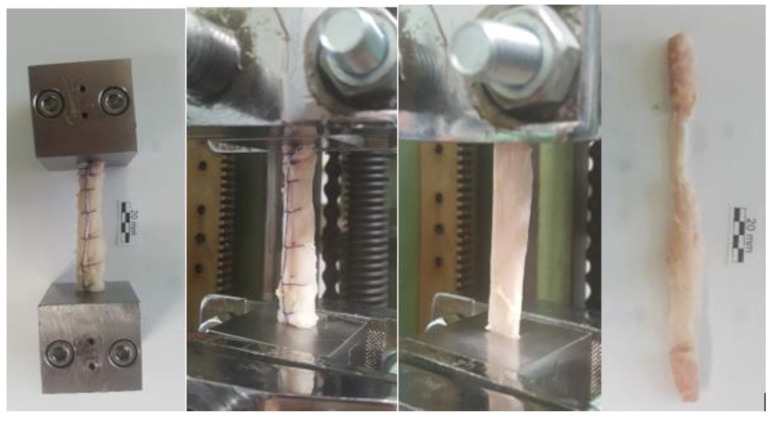
Graft preparation and testing on a mechanical testing machine.

For each graft pair, one was modified using the above suture technique to convert the tendon into a cylindrical structure. The grafts were then positioned uniformly for testing. Specifically, the bone segment was set in the graft holder right up to the junction between the bone and tendon sections. This precise positioning was consistently employed throughout all tests on the mechanical tensile tester (Figure 2).

**Figure 3:**
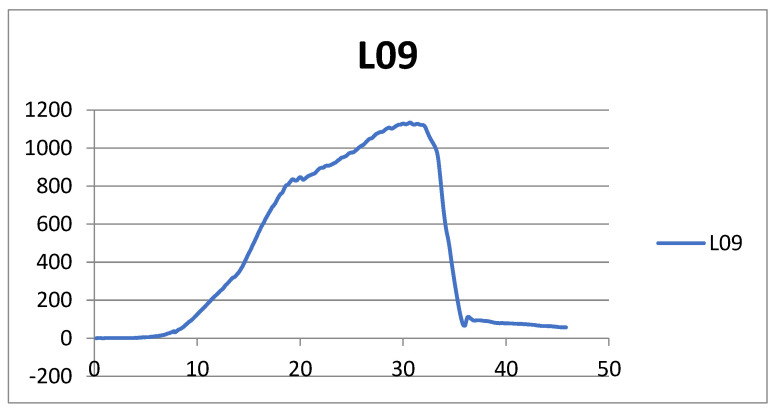
Graphic representation of the force in N/mm^2^ at which the graft ruptured.

Testing was done to determine the force required for the modified graft to mechanically tear, expressed in N/mm^2^, compared to classically prepared bone-tendon-bone grafts. The force in N/mm^2^ at which the graft ruptured was recorded for each sample (Figure 3). Clinical testing was initiated after the conclusion that BTB grafts retained their strength even after tubularization and classically processed grafts.

### 2.3. The Clinical Part of the Research

The second part of the research consisted of a prospective randomized clinical trial conducted at the Clinic for Orthopedic Surgery and Traumatology of the Clinical Center of Vojvodina in Novi Sad after receiving approval from the Ethics Committee of the University Clinical Center of Vojvodina. The study involved 120 patients who underwent operative reconstruction of the anterior cruciate ligament (ACL) of the knee, and the recruitment of patients lasted from March 2020 to November 2021.

The clinical procedure used in this study is part of the standard health service provided to patients from admission to the hospital until discharge. Each patient was informed about the research’s purpose, methodology, examinations, and measurements. 

Criteria for inclusion in the study:

- Individuals of both genders aged 16 to 45 years.
- Diagnosed isolated anterior cruciate ligament knee rupture.
- Signed informed consent and agreement to participate in the research by the patient.

Criteria for exclusion from the study:

- Patients undergoing a repeat anterior cruciate ligament surgery.
- Patients with additional injuries to the knee, including degenerative or rheumatoid processes, in addition to the anterior cruciate ligament rupture.
- Patients who developed severe general surgical complications during the study.
- Those who no longer wish to participate in the study without being obligated to explain their decision.

The standard procedure for arthroscopic reconstruction of the ACL was performed in the operating room under spinal or general endotracheal anesthesia. The patient was placed in a supine position with the leg on an arthroscopic holder, and the operations were performed with the use of a tourniquet.

The patellar tendon is accessed through a vertical skin incision from the middle of the patella to the lower part of the tibial tubercle, with the knee flexed. By separating the skin flaps, the paratenon is exposed and longitudinally incised to reveal the entire width of the patellar tendon. Using an oscillating saw, bone blocks are formed on the tibia and patella, measuring 25 mm in length and 10 mm in width. The middle third of the patellar ligament with the corresponding bone blocks is detached, and excess soft tissue is removed.

To ensure a balanced and unbiased distribution of participants into the two groups, we employed a simple randomization technique. Before the surgery, sealed opaque envelopes containing allocation cards (either ‘Tubularized’ or ‘Standard’) were prepared by a research assistant not involved in the operative procedures or outcome assessment. As each patient was enrolled and met the inclusion criteria, an envelope was drawn randomly and opened by the operating surgeon, determining the graft preparation method for that particular patient. This approach ensured that each participant had an equal chance of being assigned to either of the groups, and the allocation sequence remained concealed until the point of intervention.

**Figure 4:**
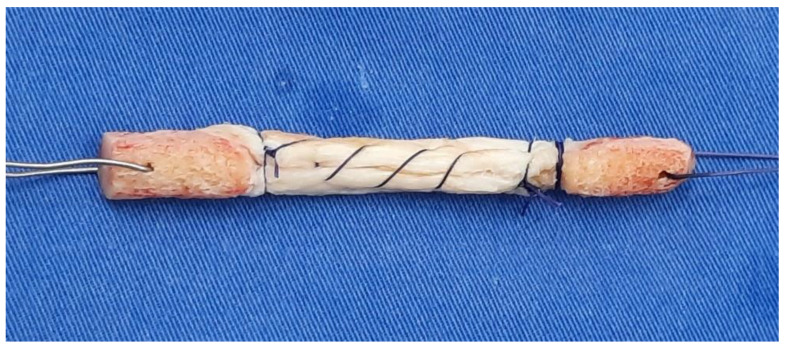
Tubularization of the graft during the operation.

Tubularization involved suturing the edges of the graft to form a tube shape from a flat ligament (Figure 4). This modification increased the cross-sectional area to 71–81 mm^2^, approximating the cross-sectional values of the native ACL. The graft was then inserted into the tunnels and fixed with cannulated titanium screws. 

All patients followed the same rehabilitation program [22]. The clinical examination of each patient included preoperative and postoperative assessments using the Tegner, Lysholm, and IKDC scoring scales for the knee [23].

Data processing was performed using SPSS for Windows 20 program, which operates under the Microsoft Windows environment. The assumption of normal distribution of numerical feature results was examined using the Kolmogorov-Smirnov test. To compare the average postoperative scores (IKDC, Tegner, Lysholm, Lachman) between the two groups of patients (classical and tubularized ligament), the χ^2^ test for categorical data was utilized, along with the non-parametric Mann-Whitney U test due to the non-normal distribution of result data. Moreover, preoperative, and postoperative results on functional tests within both groups were compared using the Wilcoxon rank test, as the data were dependent samples. Additionally, to compare the force during ligament tearing and maximum elongation of ligaments between the two groups, the t-test for independent samples was employed, considering the data was normally distributed.

## 3. Results

### 3.1. Experimental Results

The independent samples t-test results showed statistically significant differences between the classically prepared and tubularized grafts regarding the average measured force during ligament rupture (t [8] = −3.925, *p* = 0.001). Namely, ligaments prepared using the tubularized surgical technique (M = 1309.50, SD = 325.91) have statistically significantly higher values of measured force during rupture compared to ligaments prepared using the classical method (M = 775.70, SD = 280.63) (Table 1).

The results of the independent samples t-test indicate statistically significant differences between the classically prepared and tubularized grafts concerning the average maximum elongation during ligament rupture (t [8] = −2.142, *p* = 0.046). Specifically, ligaments prepared using the tubularized surgical technique exhibit significantly higher maximum elongation values during rupture (M = 28.70, SD = 6.85) compared to ligaments prepared using the classical method (M = 22.81, SD = 5.36) (Table 2).

### 3.2. Functional Results

The research sample comprised 96 male and 24 female patients, with equal representation of genders in both groups. The T-test was applied to assess the age difference between the two patient groups, and the results showed no statistically significant difference in age (t (118) = −0.6877, *p* = 0.493). Moreover, the groups were comparable in terms of observed anthropometric characteristics. The Mann-Whitney U test revealed a statistically significant gender difference in body height, body weight, and BMI in the total sample. However, no statistically significant differences were observed when comparing the two groups of male or female subjects or both groups combined (Table 3). The only distinction between the groups was in the preparation of the BTB (bone-patellar tendon-bone) graft.

Clinical parameters were monitored preoperatively and 12 months postoperatively and included examination using the Tegner, Lysholm and IKDC knee scores. Non-parametric chi-squared test was used to determine the relationship between variables with categorical data. This involved creating cross-tabulations when the prerequisites for the chi-squared test were not satisfied.

Regarding the IKDC Score, it was found that before the operation, no patients exhibited normal knee findings, while nearly normal findings were present in 24.2% of patients, abnormal findings in 35%, and severely abnormal findings in 40.8%. After the operation, normal knee findings were observed in 70% of patients, nearly normal in 22.5%, and abnormal in 7.5%, and no patients had severe abnormalities. However, there was no significant correlation found between patient groups and the IKDC questionnaire scores postoperatively (χ^2^ = 0.313, *p* = 0.855).

As for the Tegner Activity Scale, the mean Tegner scores before the surgical procedure were 5.36 ± 2.63. Following the surgery, the average scores improved to 6.40 ± 2.24. The Mann-Whitney U test did not reveal any statistically significant variations in Tegner questionnaire scores between patient groups, both before and after surgery (*p* > 0.05) (Table 4 and Table 5). Nevertheless, when analyzing the grafts prepared through classical methods, the Wilcoxon rank test demonstrated a statistically significant enhancement in the mean Tegner scores from before to after surgery (z = −2.014, *p* = 0.044) (Table 6). The median score on the questionnaire increased from 4.00 before surgery to 6.00 after surgery. Similarly, for the tubularized grafts, the Wilcoxon rank test indicated a notable difference in the mean Tegner score before and after surgery (z = −2.985, *p* = 0.003) (Table 7). The median score on the questionnaire increased from 5.50 before surgery to 6.50 after surgery.

Transitioning to the Lysholm Knee Scoring Scale, the initial Lysholm scale values ranged from 23 to 100 points, with an average of 73.60 ± 16.97. After the surgical intervention, the postoperative scores ranged from 50 to 100 points, averaging 87.52 ± 10.36. The Mann-Whitney U test indicated no noteworthy divergence in the average scores on the Lysholm scale between patient groups, both preoperatively and postoperatively (*p* > 0.05) (Table 8 and Table 9). However, for grafts prepared classically, the Wilcoxon rank test displayed statistically significant disparities in the average Lysholm score before and after surgery (z = −4.550, *p* < 0.001), with the median score increasing from 79.00 before surgery to 89.50 after surgery (Table 10). Similarly, for tubularized grafts, the Wilcoxon rank test denoted significant differences in the average Lysholm score before and after surgery (z = −5.670, *p* < 0.001), with the median score increasing from 78.00 before surgery to 90.00 after surgery (Table 11).

## 4. Discussion

Over the last decade, several papers have been published that indicate a failure range of 10% to 25% for anterior cruciate ligament (ACL) reconstruction [5,24,25]. However, determining the exact failure rate is challenging due to the lack of a well-defined criterion for “failure” in ACL reconstruction. One criterion, recurrent instability resulting from graft re-rupture, occurs in 0.7% to 8% of reconstructions [26]. Early failures, often within the first six months, are primarily attributed to technical errors, aggressive or inadequate rehabilitation, premature return to sports, or insufficient graft implantation and fixation [5]. Notably, surgical technique remains the leading cause of avoidable failure, with common errors being improper tunnel drilling and inaccuracies in graft selection, such as size, physiometry, and tension [27].

When evaluating graft healing in ACL reconstruction, it’s essential to consider intra-articular graft maturation and incorporation into bone tunnels. Abe et al. suggest that bone-tendon-bone (BTB) autografts remain immature a year post-surgery [28]. In contrast, Falconiero et al. found that hamstring and BTB grafts had matured histologically to resemble the normal ACL after 12 months [29]. Rougraf et al. propose that BTB autograft maturation takes up to 3 years after ACL reconstruction [30].

Kinguasa et al. [31] conducted a study measuring BTB autograft cross-sectional area using magnetic resonance imaging over five years post-surgery. Results indicated that the graft’s cross-sectional area rapidly increased for 3–6 months after ACL reconstruction, reaching a peak of 190% around one year, gradually decreasing thereafter and stabilizing after three years. Fukuda et al. [32] assessed graft maturation via second-look arthroscopic examinations and MRI evaluations. Their study found BTB graft maturation superior to hamstring graft maturation at arthroscopic assessment after one year and MRI evaluation two years post-surgery. Regarding the tubular design specifically, while our study did not directly measure the rate of graft resorption, its implications must be considered. The increased cross-sectional area may slow the resorption process, affecting the overall time needed for complete ligamentization. Future long-term studies are required to fully understand the impact of tubular design on graft resorption and ultimate biocompatibility.

Biomechanical studies suggest that tendon graft strength is influenced by size, with smaller dimensions leading to weaker, less stable grafts [33]. While the precise graft diameter to prevent failure isn’t entirely clear and may vary based on other factors, recent studies propose that increasing the diameter by 0.5 mm up to 10 mm benefits patients. However, there’s no clear evidence supporting grafts larger than 10 mm [34]. Conte et al. [35] found that using a quadruple-folded hamstring graft of 8 mm or more reduces failure rates, especially in patients under 20 years old [36].

Toritsuka et al. [37] compared graft cross-sectional area and tunnel filling percentage between BTB and hamstring grafts from the same patient. The primary disadvantage of BTB grafts lies in the discrepancy between the graft and bone tunnel dimensions, resulting in incomplete tunnel filling [21]. Ideally, reconstruction involves positioning bone tunnels anatomically at the ACL attachment site while matching graft and native ACL geometric properties. Although surgical procedures have improved, graft preparation methods have remained relatively unchanged.

Pujol et al. [17] modified graft preparation to match double-bundle ACL reconstruction, while Shino et al. [18] described a BTB graft reconstruction technique mimicking anteromedial and posterolateral bundles. Herbort et al. [19] attempted to correct graft-bone tunnel dimension differences by creating rectangular tunnels and corresponding bone blocks. However, these techniques overlooked the significance of the graft’s cross-sectional area. Kang et al. [20] increased cadaveric BTB graft cross-sectional area by sectioning bone blocks longitudinally and folding the ligament. Tubularization of the graft, a technique published by the Clinic for Orthopedic Surgery and Traumatology, Clinical Center of Vojvodina, involves suturing the middle third of the femoral bone block to form a tube shape [21]. This technique significantly increased the cross-sectional area to 71–81 mm^2^. Experimental comparisons between classic and tubularized grafts in our study showed higher force values and elongation during tearing for tubularized grafts.

Moralle et al. [38] published a study in 2018 aimed at evaluating whether suturing a quadruple-folded hamstring allograft improves its biomechanical properties. Similar to our research, the results showed a statistically significant increase in strength in the tubularized graft group in a pig model. Additionally, this technique increases the overall diameter of the graft and creates a uniform structure among the four bundles, preventing graft deformation. Hong et al. [39] demonstrated in a biomechanical study that the number of sutures on tendons does not affect mechanical properties concerning tearing or cyclic loading. The results of the aforementioned experimental studies indicate that tubularization through ligament suturing can enhance the process of ligamentization by increasing the strength of the graft and preventing the early failure of anterior cruciate ligament reconstruction.

In contrast to previous studies, Wang et al. [40] demonstrated that tubularization of the aponeurotic part of the semitendinosus muscle weakens the structural and mechanical properties of the graft. Similar findings were reported in other studies that examined graft properties after interfering with hamstring tendon bundles. Millett et al. [41] examined the effect of interference with hamstring tendon bundles in a cadaveric study and proved that this technique does not increase the cross-sectional area. Instead, the process itself leads collagen fibers into a suboptimal orientation, resulting in weakened graft integrity. Nicklin et al. [42] noted that interfering with tendons creates a certain angle of twisting. A twisting angle of 45° led to a 50% reduction in strength, while interference with four bundles of hamstring tendons reduced strength by 54% and tendon graft stiffness by 85%. Moore et al. [43] demonstrated tubularized BTB grafts exhibited greater tissue elongation during preconditioning. However, there was no statistically significant difference in stiffness between tubularized and flat grafts, nor did tubularization affect graft laxity.

The results of our study highlight the considerable improvement in the functional outcome of patients after ACL reconstruction as evidenced by significant improvements in the IKDC, Tegner, and Lysholm knee scores 12 months postoperatively.

Regarding the IKDC score, our findings indicate a significant shift from preoperative scores, wherein none of the patients showed normal knee findings, to postoperative results, where a majority (70%) had normal findings. This shift showcases the overall success of the procedure in restoring the functional status of the knee. Comparatively, in the study by Galan et al. [44], five years postoperatively, 82% of the patients were able to undertake high-level physical activities with an overall IKDC rating of A in 59.79% of patients. Schulz et al. [45] found that 23.6% of knees were graded as normal (A) and 41.8% as nearly normal (B). In the Struewer et al. study [46], two years post-ACL reconstruction, 37.3% of patients had an IKDC grade of A.

Considering the Tegner Activity Scale, our study found that postoperative mean scores improved from 5.36 to 6.40. Comparatively, Galan et al. [44] reported a slight decrease in median Tegner scores from 9 pre-injury to 8 at five years postoperatively. Schulz et al. [45] noted a mean loss of 0.82 index points from their pre-injury index. Struewer et al.’s results [46] reflected a minor drop from 5.8 before injury to 5.4 two years postoperatively. Notably, there might be an inevitable decline in activity levels from the pre-injury baseline across multiple studies. Nevertheless, our results show a promising improvement 12 months postoperatively.

Lastly, in terms of the Lysholm Knee Scoring Scale, our study demonstrated a significant improvement in scores from an average of 73.60 preoperatively to 87.52 postoperatively. Galan et al. [44] observed an enhancement in scores from an average of 64 preoperatively to 91 at five years postoperatively. Schulz et al. [45] reported an average Lysholm score of 89 points at their follow-up, while Struewer et al. [46] showed a slight decline from 95.7 before injury to 92.4 two years after surgery. This data suggests that while the postoperative scores in our study are comparable to those of Schulz et al. [45], patients in the Galan et al. study [44] fared better, potentially due to the extended 5-year postoperative period allowing for prolonged recovery and rehabilitation.

It’s also worth noting that the studies mentioned have varying follow-up periods ranging from 12 months (our study) to 5 years. Longer follow-ups might provide more insight into the durability of the surgical results and the long-term function of the reconstructed ACL.

The effectiveness of postoperative rehabilitation is critical for ensuring optimal outcomes following ACL reconstruction. According to a recent study, a 6-week eccentric-oriented strength training during the late-stage ACL-rehab phase demonstrated superior leg strength and jump performance for professional team sport athletes compared to traditional strength training [47]. Such findings suggest that the integration of eccentric-oriented strength training, potentially coupled with the benefits of the tubularized graft, might offer a comprehensive approach to expedite recovery and return to sports. Additionally, the biomechanical response of the graft during rehabilitation exercises is of utmost importance. With the modification of the BTB graft into a tubular design, understanding its interaction with dynamic forces becomes vital. As highlighted in this study, integrating eccentric training, specifically using flywheel mechanisms, enhanced leg strength and jump performance in athletes. The design of the graft, combined with the type of rehabilitation exercise, could play a synergistic role in determining the biomechanical response of the graft during rehab exercises.

The role of biophysics in the realm of rehabilitation has garnered attention in recent years. Emerging research emphasizes the importance of understanding the biophysical processes underpinning the macroscopic effects of treatments for musculoskeletal disorders (MSD) [48]. Techniques such as shock wave therapies, low-level laser therapy, and pulsed electromagnetic fields have been investigated from a biophysical perspective to enhance regenerative rehabilitation. Considering the process of graft ligamentization and the interactions of the graft with surrounding tissues, the biophysical properties of the graft and its response to different rehabilitative stimuli could be crucial. For instance, the tubular design of the graft may have specific biophysical properties that affect its interaction with rehabilitative treatments. Recognizing these interactions can inform rehabilitation protocols, ensuring the graft is subjected to optimal conditions that favor its integration and function within the knee.

However, it’s worth noting that while the integration of a biophysical-based approach in rehabilitation has shown promise, existing literature is still inconclusive, with clear treatment protocols yet to be established. The interplay between the graft’s design, its biophysical properties, and the dynamic forces exerted during rehabilitation is an avenue warranting further research.

## 5. Conclusions

The experimental findings reveal that suturing the graft does not compromise biomechanical properties. Furthermore, tubularization of the bone-tendon-bone autograft markedly elevates the force necessary for rupture and the maximum elongation at the point of rupture. From a clinical perspective, comparing operative outcomes with objective scores indicates no notable difference between operations using tubularized BPTB grafts and those using classically prepared grafts; both methods produce equivalent results on objective scores.

The experimental results underscore the potential of graft tubularization through suturing to enhance ligamentization and prevent early ACL reconstruction failure. However, challenges persist due to in vitro testing limitations and the inability to fully replicate in vivo conditions. Future research should address these limitations to refine graft preparation techniques and optimize ACL reconstruction outcomes.

In conclusion, ACL reconstruction has shown significant improvements in the functional outcomes across various studies. While our results are encouraging, especially in the short-term postoperative period, continued research and longer-term follow-ups are essential to fully understand the longevity and functional outcomes of the procedure.

## Figures and Tables

**Table 1:** Comparison of two groups in terms of average force during graft rupture.

Parameter	Group	N	M	SD	t	*p*
**Measured force during ligament rupture**	classic	10	775.70	280.63	−3.925	0.001
	tubularized	10	1309.50	325.91		

**Table 2:** Comparison of two groups in terms of maximum elongation before graft rupture.

Parameter	Group	N	M	SD	t	*p*
**Maximum elongation before graft rupture**	classic	10	22.81	5.36	−2.142	0.046
	tubularized	10	28.70	6.85		

**Table 3:** Mann-Whitney U test for anthropometric characteristics.

	Gender		Male		Female		Both Groups	
	Z	*p*	Z	*p*	Z	*p*	Z	*p*
Height	6.08514	<0.00001	0.71078	0.4777	0.72169	0.47152	0.25456	0.80258
Weight	6.04906	<0.00001	0.07694	0.93624	0.28868	0.77182	−0.1024	0.92034
BMI	3.99225	0.00006	0.1722	0.86502	−0.2598	0.79486	0.13909	0.88866

**Table 4:** Mann-Whitney U test—differences between the two groups of patients regarding the Tegner score before surgery.

Tegner before Surgery	N	Mean Rank	Mann-Whitney U	Wilcoxon W	Z	*p*
**classic**	60	60.44	1796.500	3626.500	−0.019	0.985
**tubularized**	60	60.56				

**Table 5:** Mann-Whitney U test—differences between the two groups of patients regarding the Tegner score after surgery.

Tegner after Surgery	N	Mean Rank	Mann-Whitney U	Wilcoxon W	Z	*p*
**classic**	60	57.41	1614.500	3444.500	−0.988	0.323
**tubularized**	60	63.59				

**Table 6:** Comparison of Tegner score values before and after surgery in subjects where the classic surgical technique for ligament reconstruction was used.

Tegner	Mdn	z	*p*
**before surgery**	4.00	−2.014	0.044
**after surgery**	6.00		

**Table 7:** Comparison of Tegner score values before and after surgery in subjects where the tubularized surgical technique for ligament reconstruction was used.

Tegner	Mdn	z	*p*
**before surgery**	5.50	−2.985	0.003
**after surgery**	6.50		

**Table 8:** Mann-Whitney U test—differences between the two groups of patients regarding the Lysholm score before surgery.

Lyscholm before Surgery	N	Mean Rank	Mann-Whitney U	Wilcoxon W	Z	*p*
**classic**	60	58.22	1663.000	3493.000	−0.722	0.470
**tubularized**	60	62.78				

**Table 9:** Mann-Whitney U test—differences between the two groups of patients regarding the Lysholm score after surgery.

Lyscholm after Surgery	N	Mean Rank	Mann-Whitney U	Wilcoxon W	Z	*p*
**classic**	60	62.98	1651.000	3481.000	−0.784	0.433
**tubularized**	60	58.02				

**Table 10:** Comparison of Lysholm score values before and after surgery in subjects where the classic surgical technique for ligament reconstruction was used.

Lyscholm	Mdn	z	*p*
**before surgery**	79.00	−4.550	<0.001
**after surgery**	89.50		

**Table 11:** Comparison of Lysholm score values before and after surgery in subjects where the tubularized surgical technique for ligament reconstruction was used.

Lyscholm	Mdn	z	*p*
**before surgery**	78.00	−5.670	<0.001
**after surgery**	90.00		

## Data Availability

The data presented in this study are available on request from the corresponding author. The data are not publicly available due to privacy reasons.
